# Present in Body or Just in Mind: Differences in Social Presence and Emotion Regulation in Live vs. Virtual Singing Experiences

**DOI:** 10.3389/fpsyg.2019.00778

**Published:** 2019-04-10

**Authors:** Daisy Fancourt, Andrew Steptoe

**Affiliations:** Department of Behavioural Science and Health, University College London, London, United Kingdom

**Keywords:** music, emotion regulation, emotions, technology, social

## Abstract

Over the past two decades, many musical experiences have become mediated by digital technology, including the distribution of music online, the generation of new content and participation in virtual musical experiences. However, whether virtual musical experiences lead to different experiences of social presence or differential use of emotion regulation strategies (ERSs) compared to live musical experiences remains un-researched. We compared the experiences of 1,158 singers in a virtual choir (VC) with the experiences of 1,158 singers from a live choir using propensity score matching based on a range of demographic, social and musical factors. Participants in VCs reported a slightly greater feeling of social presence than participants in live choirs [*t*(1157) = -19.85, *p* < 0.002]. They also made less use of overall ERSs [*t*(1157) = 3.10, *p* = 0.002], avoidance strategies [*t*(1157) = 4.51, *p* < 0.001], and approach strategies [*t*(1157) = 3.34, *p* < 0.001]. However, they made greater use of self-development strategies [*t*(1157) = -3.11, *p* = 0.002]. Social presence was associated with greater use of all ERSs. This study showed that although a sense of social presence in a choir is not reduced by engagement in VCs compared to live choirs, there is a lowered use of ERSs when engaging in VCs. However, as the difference in use of ERSs is relatively modest, virtual musical experiences may still have a role to play in supporting those who cannot engage in live experiences such as people who are socially isolated.

## Introduction

Over the past decade, there has been a rise in the availability of virtual cultural experiences, from visiting national parks, to exploring archeological sites, touring monuments, interacting with heritage buildings, collaboratively contributing to digital art, and experiencing other cultures (Gaitatzes et al., 2001; Fritz et al., 2005; Carrozzino and Bergamasco, 2010; Jung et al., 2016; Serafin et al., 2016; Efstratios et al., 2018; Wolf et al., 2018). In virtual cultural experiences, the role of virtual reality is generally either to provide visitors with a chance to visit other locations (such as cultural heritage sites or concert halls); to show reconstructions of artworks, events or sites; to provide guidance, education or storytelling; or to give visitors a chance to engage themselves in a re-enactment or activity (such as conducting a virtual orchestra) (Carrozzino and Bergamasco, 2010; Bellini et al., 2018). Virtual cultural experiences can be classified according to the level of immersion they provide. Desktop virtual reality provides 2D virtual experiences (VEs); augmented reality combines live with 2D or 3D VEs, providing an overall hybrid; and immersive reality aims for the 3D complete embodiment (Carrozzino and Bergamasco, 2010). All of these can be subcategorized into whether they are abstract VEs (such as information landscapes), non-realistic VEs (where abstract and realistic elements are co-presented), realistic VEs (either modeled or digital), or photo-realistic VEs (which are hardly distinguishable from their live counterparts) (Carrozzino and Bergamasco, 2010). They can also be further split into how many sensory channels they stimulate (how multimodal they are). These different factors combine to affect the overall level of interaction and immersion provided by a VE, with these two factors ultimately constituting how ‘present’ we feel when engaging in the VE (Carrozzino and Bergamasco, 2010; Bellini et al., 2018).

One virtual activity growing in popularity is singing in a virtual choir (VC). These choirs use the internet as a participatory platform for individuals to get involved in online crowd-sourced music-making (Literat, 2012). Studies of VCs have shown their potential in enhancing singing education, helping to achieve more complex musical performances, and encouraging greater involvement in group singing (Blackburn and McGrath, 2014; Payen, 2014). However, what the psychological impact of the experience is on individuals and how this compares to the experience of singing in a live choir remains under-explored. VCs are generally ‘desktop’ VEs that strive to recreate ‘non-realistic’ VEs in the final product (where real videos of singers are blended together with abstract design elements). They mimic the multi-voice collaborative ensemble of a live choir but in an asynchronous performance. As a result, the setting of a VC is both singular and theoretical: participants record their performances in their own individual physical localities, and then the performance is combined and presented back in cyberspace. So in this respect they differ from the human encounters in physical and social settings that underpin the experience of live choirs (Small, 1998). Further, in the actual desktop engagement with a VC, there is low sensory engagement, generally consisting of a computer monitor and speakers. So VCs are technically at the low end of both the interaction and immersion spectra (Carrozzino and Bergamasco, 2010). A key question, therefore, is whether these low levels of interaction and immersion ultimately mean that participants feel a low level of ‘presence’ when engaging in a VC and how this affects their response to the activity.

‘Presence’ can be categorized into spatial presence (the feeling of being in a particular location) and social presence (the feeling of being with others) (Willans et al., 2016). Presence has been conceptualized as a cognitive feeling and has been argued to be inherently bound with emotional responses (Meehan et al., 2002; Riva et al., 2004). This is supported by the ‘enaction’ paradigm, which proposes that emotional responses are embodied; generated by the dynamic interaction of humans in their environment (Varela, 1993; Colombetti and Thompson, 2008). Studies in VEs have shown that presence and emotional responses can co-vary, both in 3D immersive reality and desktop 2D virtual reality (Meehan et al., 2002; Riva et al., 2007; Bouchard et al., 2011; Gorini et al., 2011; Bellini et al., 2018). However, how the low levels of interaction and immersion involved in a VC affect perceptions of presence and how this is linked with emotional responses when singing remains unknown. Therefore, this study compared responses to live and VC singing amongst a large sample (*n* = 2,316) of adults.

Specifically, we focused on the emotional regulation strategies employed when singing rather than the emotional responses themselves. The reason for this was that emotional responses to singing have been shown to be diverse, affected by a broad range of factors including specific features in the music itself and individual responses to that music (e.g., personal memories evoked, visual images conjured and cultural resonance) (Juslin and Västfjäll, 2008). So in the absence of being able to standardize emotional responses to singing (and not wishing to manipulate the singing experience in a way that could become artificial), we focused not on emotional outcome but on an upstream determinant of that outcome: the emotion regulation strategies (ERSs) (van Goethem and Sloboda, 2011). ERSs are mental processes used to regulate emotions. The literature on emotion regulation theory and ERSs has emerged from broader literature on coping (especially emotion focused coping) (Lazarus and Folkman, 1984), but over the last 25 years it has grown into its own area of research, with coping distinguished by is predominant focus on reducing negative affect and its emphasis on much longer periods of time (e.g., coping with bereavement) (Gross, 2015). In comparison, ERSs allow us to consider the much more immediate effects of events and experiences. Previous research has suggested that individuals vary in the extent to which they use ERSs when engaging in creative activities including singing, as well as varying in the type of strategies they employ and the extent to which they employ them (Fancourt et al., 2019). Three broad types of ERSs have been identified when engaging in artistic creative activities: avoidance strategies (such as distraction, suppression or detachment from negative or stressful emotions), approach strategies (such as acceptance, reappraisal, and problem solving), and self-development strategies (such as enhanced self-identity, improved self-esteem, and increased agency) (Fancourt et al., 2019). In this study, we tested three hypotheses. We hypothesized that, due to the low levels of interaction and immersion involved in VC, (i) individuals engaging in VCs would feel a lower level of social presence than those engaging in a live choir; (ii) individuals engaging in VCs would make less use of ERSs (both generally and in relation to all three ERS categories); (iii) levels of ERSs used would positively correlate with levels of perceived social presence.

## Materials and Methods

### Participants

**FIGURE 1:**
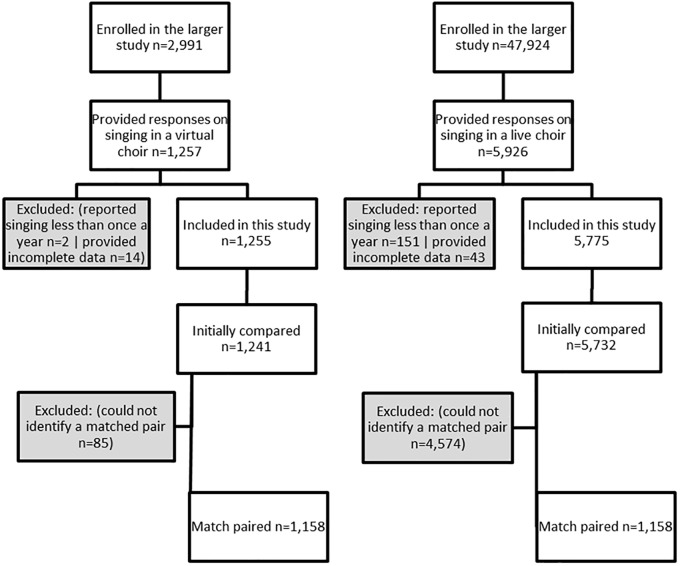
Participant selection for involvement in study analyses.

We collected data from participants enrolled in the program Virtual Choir 5.0: a global VE project that runs every few years (see section “Procedure”). Of 2,991 adults (age 18+) who enrolled on a larger study, 1,257 provided data on experiences of singing in a VC. We compared the responses of this VC group with data in *The Great British Creativity Test*: a citizen science project that includes responses from 47,924 adults (age 18+) on engagement in creative activities. Of these participants, 5,775 provided data on experiences of singing in a live choir. To account for differences in baseline characteristics between the two samples, we used propensity score matching to create a propensity-matched cohort of a total of 2,316 people (see Figure 1). For both studies, inclusion criteria were that participants had to be above the age of 18, and have engaged in singing in the past year. Both studies were approved by the University College London Research Ethics Committee, all participants gave written informed consent, and the study complied with APA ethical standards.

### Procedure

Virtual Choir 5.0 is a VC program run by composer Eric Whitacre. According to this VC platform, singers rehearse their part in the choir through singing along to a recording of the piece by a backing choir and watching a video of a conductor, who can also provide a lesson in singing the piece. Singers then film themselves singing their individual parts and submit them online. Singers can geo-tag their video submissions to indicate their geographic location so that others can see the ‘place’ involved, even though the choir itself is fundamentally ‘placeless.’ Each individual’s recording can be viewed by clicking on their geotag. The videos are also collated and edited before being combined in a network animation that does not graphically emulate a live choir but provides an abstract interpretation of a choir. Videos from Virtual Choir 5.0 can be viewed at https://www.youtube.com/channel/UCrjq25xbdEiL8jH28FR38Ow. Throughout the process, participants can connect with one another through a bespoke social media platform. For this study, following involvement in the VC, participants were asked to complete an online questionnaire about their experiences. This questionnaire took approximately 10 min to complete. The comparable data about engagement in live choirs came from the *Great British Creativity Test*: an online Citizen Science project led by the BBC that asks about engagement with creative activities and ERSs when engaging in these activities. This questionnaire took approximately 20 min to complete (Fancourt et al., 2019).

### Measures

For these analyses, both data sets included the Emotion Regulation Strategies for Artistic Creative Activities scale (ERS-ACA) which includes a general factor as well as avoidance, approach, and self-development subscales, all scored from 1 to 5 with higher scores indicating greater use of the strategy (Fancourt et al., 2019). Avoidance strategies (e.g., distraction, suppression or detachment from negative or stressful emotions) are measured with questions such as “when engaging in an artistic creative activity…I can block out any unwanted thoughts or feelings,” “…I can shake off any anxieties in my life,” and “…it helps me to disengage from things that are bothering me.” Approach strategies (e.g., acceptance, reappraisal and problem solving) are measured with questions such as “…it helps me refocus on what matter in my life,” “…it helps me to come to terms with my own emotions,” and “…it helps me to understand my own feelings on things that are on my mind.” Self-development strategies (e.g., enhanced self-identity, improved self-esteem, and increased agency) are measured with questions such as “…I feel more confident in myself,” “…it boosts my self-esteem,” and “…it gives me a sense of purpose.” The overall scale performed in line with its validation, with an overall Cronbach’s alpha of 0.94 for the entire sample (0.94 for VC participants and 0.93 for live choir participants), and subscale alphas of 0.88, 0.88, and 0.85.

Social presence was measured through three items (all rated from strongly disagree -1 to strongly agree -5). These items were “When singing in a [virtual/live] choir: I feel a part of something bigger/I feel a sense of connection to other people (even people I don’t know) who are doing the same activity/I feel like I’m part of a community.” The average was taken of the three responses. The item had a Cronbach’s alpha of 0.85 for the entire sample (0.76 for VC participants and 0.86 for live choir participants).

### Statistical Analysis

To create a matched cohort of live vs. VC participants, we calculated the propensity score (logit model) for each individual based on demographic variables (age, sex, ethnicity, and employment status), social variables (whether an individual lived alone and perceived loneliness) and singing experience (number of years singing and frequency of singing). We then used nearest available Mahalanobis metric 1-to-1 matching method without replacement, using a caliper size of 0.25 using the Stata module PSMATCH2 (Leuven and Sianesi, 2018). Success of the propensity score matching was assessed using Rubin’s *B* < 25 (*B* = 4.6), Rubin’s R of 0.5-2 (*R* = 1.1) and a percentage bias of <10% for each covariate (bias = 0.2–4.0%) (Rubin, 2001; Morgan, 2018).

For unmatched data (prior to the matching procedure), differences between groups (virtual vs. live choir participation) were analyzed using independent *t*-tests and χ^2^ tests. For matched data, differences between groups were analyzed using paired *t*-tests, Wilcoxon signed-ranks test, and McNemar’s test. Pearson’s pairwise correlations were used to explore associations between presence and ERSs. To account for a sense of social presence, we categorized social presence into low (1–3) vs. high (4–5) and analyzed a sub-set of the sample for whom both participants within the pair scored low or high respectively. This provided a reduced sample of 66 pairs in which both partners scored low and 516 pairs in which both partners scored high. We then re-ran analyses of ERSs using this stratified sub-sample. Analyses were carried out using Stata v14 (StataCorp, College Station, TX, United States).

## Results

### Participant Characteristics Before and After Matching

**FIGURE 2:**
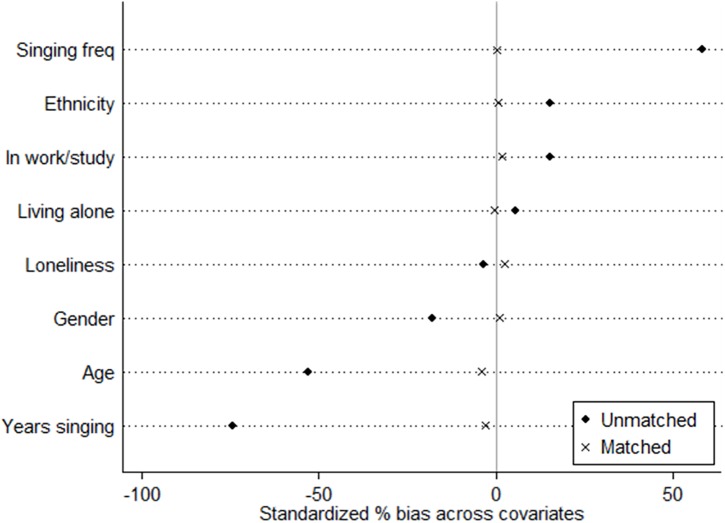
Standardized bias (%) across covariates in the propensity score before and after matching.

Demographic characteristics of participants before and after propensity score matching are shown in Table 1. Before matching, a greater number of participants in the VC were younger, female, white and in work or study. They had also typically been singing for fewer years but sang more frequently now. After matching, participants were well-matched on all characteristics (see Figure 2).

**Table 1:** Demographic characteristics of participants in virtual and live choirs before and after propensity score matching.

	Before matching			After matching		
		
	Virtual choir (*n* = 1,241)	Live choir (*n* = 5,732)	*p*	Virtual choir (*n* = 1,180)	Live choir (*n* = 1,180)	*p*
Age (mean years, SD)	36.6 ± 15.3	44.6 ± 14.4	**<0.001**	37.0 ± 15.4	37.6 ± 15.3	0.20
Gender (female)	865 (69.7%)	3,507 (61.2%)	**<0.001**	800 (69.2%)	806 (69.6%)	0.79
Ethnicity (white)	998 (84.4%)	5,130 (89.5%)	**<0.001**	984 (85.0%)	987 (85.2%)	0.90
In work/study	1,098 (88.5%)	4,779 (83.4%)	**<0.001**	1,024 (88.4%)	1,017 (87.8%)	0.65
Living alone	218 (17.6%)	905 (15.8%)	0.12	206 (17.8%)	207 (17.9%)	<0.99
Years singing			**<0.001**			0.10
<10 years	494 (39.5%)	1,108 (19.3%)		445 (38.4%)	450 (38.9%)	
10–19 years	400 (32.3%)	939 (16.4%)		381 (32.9%)	343 (29.6%)	
20–39 years	236 (19.0%)	2,016 (35.2%)		225 (19.4%)	250 (21.6%)	
40+ years	110 (8.9%)	1,669 (29.1%)		107 (9.2%)	115 (9.9%)	
Freq of singing			**<0.001**			0.77
<once a week	14 (1.1%)	637 (11.1%)		12 (1.0%)	21 (1.8%)	
Every week	303 (24.5%)	2,204 (38.5%)		291 (25.1%)	276 (23.8%)	
Every day	922 (74.4%)	2,891 (50.4%)		855 (73.8%)	861 (74.4%)	
Loneliness			0.56			0.73
Hardly ever	544 (43.9%)	2,453 (42.8%)		500 (43.2%)	504 (43.5%)	
Sometimes	536 (43.2%)	2,477 (43.2%)		506 (43.7%)	519 (44.8%)	
All the time	160 (12.9%)	802 (14.0%)		152 (13.1%)	135 (11.7%)	


**Bold indicates *p* < 0.05.**

#### Hypothesis 1: Differences in Social Presence Between Virtual and Live Choirs

Comparisons in self-rated social presence between virtual and live choirs showed that, contrary to our hypothesis, participants in VCs in fact felt more socially present (mean: 4.20, *SE*: 0.02) than participants in live choirs (mean 3.46, SE 0.03) (see Table 2).

**Table 2:** Paired *t*-tests showing the use of ERSs and sense of social presence between matched participants in virtual and live choirs.

	Virtual choir (*n* = 1,158)	Live choir (*n* = 1,158)	
			
	Means ± SE	Means ± SE	
**Social presence**
Sense of being part of a group	4.20 ± 0.02	3.46 ± 0.03	*t*(1157) = -19.85, ***p* < 0.002**
**Emotion regulation strategies**	
Overall use of ERSs	3.55 ± 0.02	3.63 ± 0.02	*t*(1157) = 3.10, ***p* = 0.002**
Use of avoidance strategies	3.64 ± 0.02	3.77 ± 0.02	*t*(1157) = 4.51, ***p* < 0.001**
Use of approach strategies	3.24 ± 0.02	3.41 ± 0.02	*t*(1157) = 3.34, ***p* < 0.001**
Use of self-development strategies	3.78 ± 0.02	3.69 ± 0.02	*t*(1157) = -3.11, ***p* = 0.002**


**Bold indicates *p* < 0.05.**

#### Hypothesis 2: Differences in ERSs Between Virtual and Live Choirs

Comparisons in use of ERSs between virtual and live choirs confirmed our hypothesis that participants in VCs made less use of ERSs overall than participants in live choirs (see Table 2). Specifically, they made less use of avoidance strategies and approach strategies. However, contrary to our hypothesis, participants in VCs made more use of self-development strategies.

#### Hypothesis 3: Interactions Between ERSs and Social Presence

Correlations support our third hypothesis, showing a strong overall correlation between social presence and use of ERSs, with moderate correlations between social presence and both avoidance and approach strategies, and strong correlations between social presence and use of self-development strategies. Comparable findings were shown in the entire sample and also within subgroups of VC and live choir (see Table 3).

**Table 3:** Pearson’s correlations of use of ERSs and social presence.

	Social presence		
	
	Overall (*n* = 2,316)	Virtual choir (*n* = 1,158)	Live choir (*n* = 1,158)
Overall use of ERSs	*r* = 0.44, ***p* < 0.001**	*r* = 0.47, ***p* < 0.001**	*r* = 0.55, ***p* < 0.001**
Use of avoidance strategies	*r* = 0.30, ***p* < 0.001**	*r* = 0.34, ***p* < 0.001**	*r* = 0.41, ***p* < 0.001**
Use of approach strategies	*r* = 0.32, ***p* < 0.001**	*r* = 0.45, ***p* < 0.001**	*r* = 0.38, ***p* < 0.001**
Use of self-development strategies	*r* = 0.56, ***p* < 0.001**	*r* = 0.48, ***p* < 0.001**	*r* = 0.64, ***p* < 0.001**


**Bold indicates *p* < 0.05.**

**FIGURE 3:**
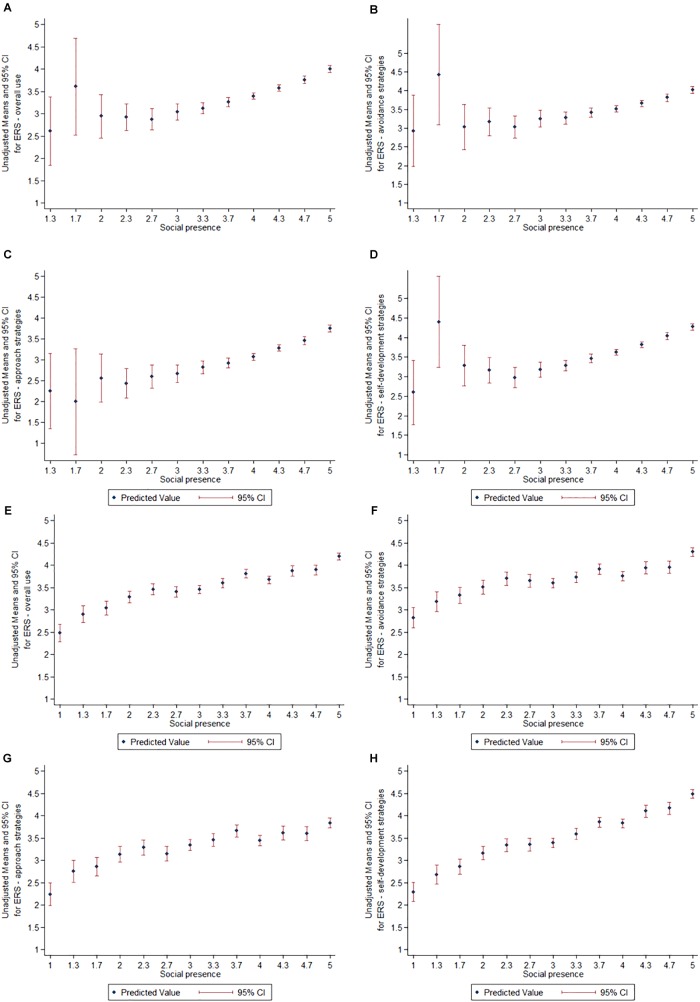
Means and 95% confidence intervals for emotion regulation strategies by social presence rating for virtual choirs **(A–D)** and live choirs **(E–H)**.

To explore this relationship further, we graphed mean ERS responses by social presence rating (Figure 3). There was a clear linear dose–response relationship between social presence and use of ERSs amongst respondents in the live choirs with relatively even confidence intervals throughout. In contrast, lower social presence ratings amongst those in the VCs had non-linear relationships with use of ERSs, with large confidence intervals.

To account for a sense of social presence, we categorized social presence into low (1–3) vs. high (4–5) and analyzed a sub-set of the sample for whom both participants within the pair scored low or high respectively. Sensitivity analyses involving pairs in which both participants reported either low social presence or high social presence showed that differential levels of use of ERSs for overall use, avoidance strategies and approach strategies between virtual and live were maintained. For self-development strategies, amongst pairs who both reported a sense of low social presence, there was no significant difference in use, while for pairs who reported a sense of high social presence, those in VCs in fact reported lower use of self-development strategies. However, it should be noted that the numbers in the ‘low sense of social presence’ group were very small so power may have been affected. Results from these analyses are shown in Table 4.

**Table 4:** Paired *t*-tests showing the use of ERSs stratified by low vs. high sense of social presence.

Low sense of social presence	Virtual choir (*n* = 66) Means ± SE	Live choir (66) Means ± SE	
Overall use of ERSs	2.97 ± 0.07	3.34 ± 0.09	*t*(65) = 3.16, ***p* = 0.002**
Use of avoidance strategies	3.12 ± 0.09	3.51 ± 0.09	*t*(65) = 2.97, ***p* = 0.004**
Use of approach strategies	2.65 ± 0.07	3.21 ± 0.11	*t*(65) = 4.27, ***p* < 0.001**
Use of self-development strategies	3.13 ± 0.08	3.27 ± 0.11	*t*(65) = 0.95, *p* = 0.35

**High sense of social presence**	**Virtual choir (*n* = 516) Means ± SE**	**Live choir (516) Means ± SE**	

Overall use of ERSs	3.59 ± 0.03	3.91 ± 0.02	*t*(515) = 9.55, ***p* < 0.001**
Use of avoidance strategies	3.68 ± 0.03	4.00 ± 0.03	*t*(515) = 7.89, ***p* < 0.001**
Use of approach strategies	3.29 ± 0.03	3.65 ± 0.03	*t*(515) = 8.34, ***p* < 0.001**
Use of self-development strategies	3.84 ± 0.03	4.12 ± 0.03	*t*(515) = 7.51, ***p* < 0.001**


**Bold indicates *p* < 0.05.**

## Discussion

This study explored differences in perceptions of social presence and the use of ERSs amongst singers in live and VCs. We found that, amongst matched pairs of singers, singing in a VC was associated with a higher overall perception of social presence along with a lower overall use of ERSs, including specifically a lower use of avoidance strategies (such as distraction, suppression or detachment from negative or stressful emotions), a lower use of approach strategies (such as acceptance, reappraisal, and problem solving), and a higher use of self-development strategies (such as enhanced self-identity, improved self-esteem, and increased agency). Social presence was associated with greater use of all ERSs. We found no evidence that social presence moderated the overall use of ERSs or use of approach and avoidance strategies between virtual and live choirs. But we did find a moderating effect for the use of self-development strategies, which differed depending on how much presence an individual felt whilst singing in a VC.

The finding that VCs are associated with a greater sense of social presence is in one sense surprising (and contrary to our original hypothesis) given the fact that VCs involve low levels of both interaction and immersion (Carrozzino and Bergamasco, 2010). Further, it has been found in some studies that engagement with digital technology can promote a lack of human contact and a reduced feeling of ‘belonging’ (Wilson, 2018). On the other hand, it is unlikely that people would engage with VCs unless they received a strong sense of individual or social gratification. Indeed, ethnographic studies of engagement with VCs have suggested that they can in fact lead to the development of a sense of community, a reduction in perceived isolation and the formation of specific social bonds (French, 2017). And studies of musical communities on the internet have suggested that digital social communication can have consequences for lived social worlds (Lysloff, 2003). Even if they are not technologically sophisticated, digital experiences are equally ‘real’ for participants as live experiences and can lead to the formation of real, consequential social bonds (Markham, 1998). Indeed, from a sociology perspective, VR activities in cyberspace are not poor substitutes for activities in physical space, but are simply different, leading to the suggestion that they are referred to not as ‘virtual’ spaces (implying ‘unreal’) but rather as ‘socio-mental’ spaces (Chayko, 2008). In line with this, the concept of ‘community’ has itself changed since the invention of the internet, away from referring to spatially-bounded communities to including self-defined communities of common interest (Castells, 2002). It has been proposed that these communities are in fact stronger than spatially-bounded communities as they are created not out of accidents of proximity but by choice (Jones, 1995). Live choirs are already a community of choice in that people are brought together for the shared interest in singing, but spatial community still plays a role in the selection of a choir to be a member of, and this can lead to perceptions of hierarchies in live choirs that mimic the social hierarchies in the geographical area. In contrast, VCs are entirely free from geographical considerations, thereby supporting non-hierarchical relationships that may create a particularly strong sense of social capital (Putnam, 2001). Further studies have also suggested that digital technology can reduce social isolation and enhance social connectedness and perceived social support (although effects on loneliness are unclear) (Chen and Schulz, 2016). Particularly given that our sample was matched on social variables as well as demographic and musical variables (including living status and perceived loneliness), and therefore the differences in sense of social presence appear not to be driven by individual variations in social factors, the results here suggest that VCs could be valuable interventions to explore further as a way of combatting social isolation.

Our study also found differences in use of ERSs, partly supporting our original hypothesis that VCs are associated with lower use of overall ERSs and both approach and avoidance strategies. Whether certain ERSs are more or less healthy or adaptive than others has been fiercely debated. Some studies have suggested that avoidance ERSs predict negative emotions and are therefore unhealthy or maladaptive (Aldao et al., 2010; Machell et al., 2015). However, others have suggested that effective use of any ERSs (regardless of type) is associated with better mental health (Kashdan et al., 2014), or argued that nearly every ERS can appear healthy at the surface level but can be misused (Kashdan et al., 2015). In relation to engagement in artistic creative activities such as singing, where both positive and negative emotions may be regulated by the activity, it could be argued that both avoidance and approach strategies have the potential to be beneficial (Kashdan et al., 2015). Indeed, a large body of literature has demonstrated the beneficial effects of singing and music for mental health in young children, adolescents and adults, with a wide range of both approach and avoidance ERSs appearing to play a key role (Saarikallio and Erkkilä, 2007; Saarikallio, 2011; Winsler et al., 2011). Therefore, the finding that VCs are associated with less use of ERSs (both generally and in relation to both approach and avoidance strategies) suggests a muted response when engaging in VCs. Previous studies have suggested that VCs can still lead to the experience of strong emotions. For example, an ethnographic study discussed performers reporting ‘chills’ and emotional ‘highs’ (Armstrong, 2012). However, studies of live vs. digital musical experiences have found less strong emotional responses when listening to recorded vs. live music (Bailey, 1983; Shoda et al., 2016), supporting the findings reported here. It is also of note that both those in the VCs and live choirs reported greater use of avoidance rather than approach strategies, suggesting that singing helps to distract people from worries or stressors in their daily lives more than face up to these problems.

However, contrary to our hypothesis, we found that singers in VCs make greater use of self-development strategies than singers in live choirs. There are a few possible explanations for this. First, in VCs each individual member of the choir is treated simultaneously as a group member and as a soloist. Instead of a voice merely being lost in the overall group sound, individuals in the Virtual Choir 5.0 could listen to their specific contribution in the process of submission and may therefore have felt more personal achievement. Second, singing in a VC gave individuals the chance to record and re-record their contribution until they were happy with it. This may have led to a greater sense of satisfaction with one’s performance than singing live and therefore increased one’s sense of self-esteem and confidence. Third, the decision to engage with a VC may be partly associated with a desire to improve self-confidence in singing.

Finally, we found support for our third hypothesis that there is a correlation between perceived social presence and use of ERSs. This link between presence and ERSs found across our study echoes previous literature (Riva et al., 2007), and is also echoed in studies showing the inter-relationship between emotional and social responses to music listening (Västfjäll, 2003; Juslin et al., 2008), including studies showing that experiencing music with others leads to stronger emotional responses (Liljeström et al., 2013). The finding that use of self-development strategies is moderated by sense of social presence is intuitive, as it suggests that if singers did not feel a strong sense of social connectedness when participating in the VC, they in fact felt less benefits from being involved, including feeling a lower sense of self-esteem and thereby making less use of self-development ERSs.

This study has a number of strengths including its large sample size, its direct comparison of engagement in live and VCs through matched questions across two different datasets, and its use of propensity score matching to help achieve exchangeability between the two groups. However, several limitations remain. First, this study asked people about their experiences in live and VCs so was observational rather than specifically involving experimental manipulation. On the one hand, this means that the study is arguably ecologically stronger than an experiment might have been in that it draws on people’s natural experiences of singing rather than controlled experiences. On the other hand, it remains possible that latent confounding variables that may have been imbalanced between matched pairs could have affected results, so future intervention studies are recommended. Further, in this study, we did not include a measure of physical space, so whether *spatial* aspects of presence have a different relationship with ERSs remains unknown. It should also be noted that our measure of social presence was based on just three individual self-report items. As such it may lack discriminatory rigor in relation to other aspects of presence. Future work assessing presence in more detail using validated measures is therefore recommended. We also did not include a measure of technological capability so could not assess whether those in the VC were more advanced in their use of technology than those in the live choir and therefore whether this could have acted as an additional confounding variable. However, both virtual and live choir respondents had to undertake their surveys online, so a minimum technological ability was demonstrated in both groups. Similarly, we have evidence that those who sang in the VC had also sung in live choirs as over half of participants singing since before the internet was invented, thereby showing that these participants have experience of singing in live choirs too. While we matched participants closely on a range of demographic, social and musical variables, we did not compare the response to live vs. VCs within individuals. This remains a possible extension of the findings here for future studies. Additionally, this study focused on participants in a range of live choirs, but just one VC program: Virtual Choir 5.0. How VCs involving different combinations of immersion and interaction affect presence and ERSs remains to be explore. Finally, determining causality is challenging. Although we matched participants on a wide range of background demographic, social and musical factors, it remains possible that unmeasured characteristics served as confounding variables. For example, those who selected to join a VC may have been those who have had particularly strong emotional responses to live choirs. Similarly, it remains unknown how previous perceived benefits of singing for emotion regulation might feed into the motivation to engage in singing either in live or VCs.

Over the past two decades, many musical experiences have become mediated by digital technology, including the distribution of music online, the generation of new content and the participation in digital musical experiences. However, questions concerning the nature and impact of such digital musical experiences remain broadly unanswered. This study showed that perceived social presence can still be high for virtual musical experiences, even when the technological sophistication involved is modest. Further, although there is a lowered use of ERSs when engaging in VCs, this difference is relatively small. So virtual cultural experiences may still have a role to play in supporting those who cannot engage in live experiences such as people who are socially isolated.

## Ethics Statement

University College London Research Ethics Committee. All participants gave informed consent.

## Author Contributions

DF carried out the analyses and drafted the manuscript. Both authors designed the study critically appraised the manuscript and approved it for submission.

## Conflict of Interest Statement

The authors declare that the research was conducted in the absence of any commercial or financial relationships that could be construed as a potential conflict of interest. The handling Editor declared a shared affiliation, though no other collaboration, with the authors at the time of review.

## References

[B1] Aldao A. Nolen-Hoeksema S. Schweizer S. (2010). Emotion-regulation strategies across psychopathology: a meta-analytic review. *Clin. Psychol. Rev.* 30 217–237. 10.1016/j.cpr.2009.11.004 20015584

[B2] Armstrong M. (2012). *Musicking in Cyberspace: Creating Music and Fostering Global Community through a Virtual Choir.* Available at: https://dl.tufts.edu/catalog/tufts:20724 (accessed August 23, 2018).

[B3] Bailey L. M. (1983). The effects of live music versus tape-recorded music on hospitalized cancer patients. *Music Ther.* 3 17–28. 10.1093/mt/3.1.17

[B4] Bellini N. Bergamasco M. Brehonnet R. Carrozzino M. Lagier J. (2018). Virtual cultural experiences: the drivers of satisfaction. *Symphonya. Emerg. Issues Manag.* 2 52–65. 10.4468/2018.2.5bellini.bergamasco.brehonnet.carozzino.lagier

[B5] Blackburn A. McGrath N. (2014). *Anytime, Anyplace, Anywhere: New Media and Virtual Tools Offer Constructivist Learning in Online Music Education.* Available at: https://www.learntechlib.org/primary/p/148742/ (accessed August 23, 2018).

[B6] Bouchard S. Dumoulin S. Michaud M. Gougeon V. (2011). Telepresence experienced in videoconference varies according to emotions involved in videoconference sessions. *Ann. Rev. Cyberther. Telemed.* 9 104–107. 21685654

[B7] Carrozzino M. Bergamasco M. (2010). Beyond virtual museums: experiencing immersive virtual reality in real museums. *J. Cult. Herit.* 11 452–458. 10.1016/j.culher.2010.04.001

[B8] Castells M. (2002). *The Internet Galaxy: Reflections on the Internet, Business, and Society.* Oxford: Oxford University Press 10.1093/acprof:oso/9780199255771.001.0001

[B9] Chayko M. (2008). *Portable Communities: The Social Dynamics of Online and Mobile Connectedness.* New York, NY: Suny Press.

[B10] Chen Y.-R. R. Schulz P. J. (2016). The effect of information communication technology interventions on reducing social isolation in the elderly: a systematic review. *J. Med. Internet Res.* 18:e18. 10.2196/jmir.4596 26822073 PMC4751336

[B11] Colombetti G. Thompson E. (2008). “The feeling body: Towards an enactive approach to emotion,” in *Developmental Perspectives on Embodiment and Consciousness*, eds Overton W. F. Müller U. Newman J. L. (New York, NY: Erlbaum), 45–68.

[B12] Efstratios G. Michael T. Stephanie B. Athanasios L. Paul Z. George P. (2018). “New Cross/Augmented Reality Experiences for the Virtual Museums of the Future,” in *Digital Heritage. Progress in Cultural Heritage: Documentation, Preservation, and Protection Lecture Notes in Computer Science*, eds Ioannides M. Fink E. Brumana R. Patias P. Doulamis A. Martins J. (New York, NY: Springer International Publishing), 518–527. 10.1007/978-3-030-01762-0_45

[B13] Fancourt D. Garnett C. Spiro N. West R. Mullensiefen D. (2019). How do creative activities regulate our emotions? validation of the emotion regulation strategies for artistic creative activities scale (ERS-ACA). *PLoS One* 14:e0211362. 10.1371/journal.pone.0211362 30721269 PMC6363280

[B14] French M. K. (2017). *Online Music Education: The Fuel Education Virtual Choir Project*. Available at: http://www.studiomusicatreviso.it/icnmc/library/Paper_35_2017.pdf (accessed August 23, 2018).

[B15] Fritz F. Susperregui A. Linaza M. T. (2005). “Enhancing cultural tourism experiences with augmented reality technologies,” in *Proceedings of the 6th International Symposium on Virtual Reality, Archaeology and Cultural Heritage (VAST)* (San Sebastián: Asociación VICOMTech).

[B16] Gaitatzes A. Christopoulos D. Roussou M. (2001). “Reviving the past: cultural heritage meets virtual reality,” in *Proceedings of the 2001 Conference on Virtual Reality, Archeology, and Cultural Heritage* (ACM), New York, NY, 103–110. 10.1145/584993.585011

[B17] Gorini A. Capideville C. S. De Leo G. Mantovani F. Riva G. (2011). The role of immersion and narrative in mediated presence: the virtual hospital experience. *Cyberpsychol. Behav. Soc. Network.* 14 99–105. 10.1089/cyber.2010.0100 20649451

[B18] Gross J. J. (ed.) (2015). *Handbook of Emotion Regulation*, 2nd Edn New York, NY: The Guilford Press.

[B19] Jones S. (1995). *Understanding Community in the Information Age.* California: Sage.

[B20] Jung T. Tom Dieck M. C. Lee H. Chung N. (2016). “Effects of Virtual Reality and Augmented Reality on Visitor Experiences in Museum,” in *Information and Communication Technologies in Tourism 2016*, eds Inversini A. Schegg R. (New York, NY: Springer International Publishing),621–635.

[B21] Juslin P. N. Liljeström S. Västfjäll D. Barradas G. Silva A. (2008). An experience sampling study of emotional reactions to music: listener, music, and situation. *Emotion* 8 668–683. 10.1037/a0013505 18837617

[B22] Juslin P. N. Västfjäll D. (2008). Emotional responses to music: the need to consider underlying mechanisms. *Behav. Brain Sci.* 31 559–575. 10.1017/S0140525X08005293 18826699

[B23] Kashdan T. B. Goodman F. R. Machell K. A. Kleiman E. M. Monfort S. S. Ciarrochi J. (2014). A contextual approach to experiential avoidance and social anxiety: evidence from an experimental interaction and daily interactions of people with social anxiety disorder. *Emotion* 14 769–781. 10.1037/a0035935 24749634 PMC4191827

[B24] Kashdan T. B. Young K. C. Machell K. A. (2015). Positive emotion regulation: addressing two myths. *Curr. Opin. Psychol.* 3 117–121. 10.1016/j.copsyc.2014.12.012

[B25] Lazarus R. S. Folkman S. (1984). *Stress, Appraisal, and Coping.* New York, NY: Springer Publishing Company.

[B26] Leuven E. Sianesi B. (2018). *“PSMATCH2: Stata Module to Perform Full Mahalanobis and Propensity Score Matching, Common Support Graphing, and Covariate Imbalance Testing,” Statistical Software Components S432001.* Boston, MA: Boston College Department of Economics.

[B27] Liljeström S. Juslin P. N. Västfjäll D. (2013). Experimental evidence of the roles of music choice, social context, and listener personality in emotional reactions to music. *Psychol. Music* 41 579–599. 10.1177/0305735612440615

[B28] Literat I. (2012). The work of art in the age of mediated participation: crowd sourced art and collective creativity. *Int. J. Commun.* 6:23.

[B29] Lysloff R. T. (2003). Musical community on the internet: an on-line ethnography. *Cult. Anthropol.* 18 233–263. 10.1525/can.2003.18.2.233

[B30] Machell K. A. Goodman F. R. Kashdan T. B. (2015). Experiential avoidance and well-being: a daily diary analysis. *Cogn. Emot.* 29 351–359. 10.1080/02699931.2014.911143 24800802

[B31] Markham A. N. (1998). *Life Online: Researching Real Experience in Virtual Space (Ethnographic Alternatives, No 6).* Maryland, MD: Rowman Altamira.

[B32] Meehan M. Insko B. Whitton M. Brooks F. P. Jr. (2002). Physiological measures of presence in stressful virtual environments. *ACM Trans. Graph.* 21 645–652. 10.1145/566654.566630 16167189

[B33] Morgan C. J. (2018). Reducing bias using propensity score matching. *J. Nucl. Cardiol.* 25 404–406. 10.1007/s12350-017-1012-y 28776312

[B34] Payen M. (2014). “Bringing Polychoral Composition into the Virtual Era,” in *Proceedings of the Georgia Southern University Research Symposium*, Statesboro.

[B35] Putnam R. D. (2001). *Bowling Alone: The collapse and Revival of American Community.* New York, NY: Simon and Schuster.

[B36] Riva G. Mantovani F. Capideville C. S. Preziosa A. Morganti F. Villani D. (2007). Affective interactions using virtual reality: the link between presence and emotions. *CyberPsychol. Behav.* 10 45–56. 10.1089/cpb.2006.9993 17305448

[B37] Riva G. Waterworth J. A. Waterworth E. L. (2004). The layers of presence: a bio-cultural approach to understanding presence in natural and mediated environments. *CyberPsychol. Behav.* 7 402–416. 10.1089/cpb.2004.7.402 15331027

[B38] Rubin D. B. (2001). Using propensity scores to help design observational studies: application to the tobacco litigation. *Health Serv. Outcomes Res. Methodol.* 2 169–188. 10.1023/A:1020363010465

[B39] Saarikallio S. (2011). Music as emotional self-regulation throughout adulthood. *Psychol. Music* 39 307–327. 10.1177/0305735610374894

[B40] Saarikallio S. Erkkilä J. (2007). The role of music in adolescents’ mood regulation. *Psychol. Music* 35 88–109. 10.1177/0305735607068889

[B41] Serafin S. Erkut C. Kojs J. Nilsson N. C. Nordahl R. (2016). Virtual reality musical instruments: state of the art, design principles, and future directions. *Comput. Music J.* 40 22–40. 10.1162/COMJ_a_00372

[B42] Shoda H. Adachi M. Umeda T. (2016). How live performance moves the human heart. *PLoS One* 11:e0154322. 10.1371/journal.pone.0154322 27104377 PMC4841601

[B43] Small C. (1998). *Musicking: The Meanings of Performing and Listening.* Middletown, CT: Wesleyan University Press.

[B44] van Goethem A. Sloboda J. (2011). The functions of music for affect regulation. *Music. Sci.* 15 208–228. 10.1177/102986491101500205

[B45] Varela F. J. (1993). *The Embodied Mind: Cognitive Science and Human Experience*, New Edn Cambridge, MA: MIT Press.

[B46] Västfjäll D. (2003). The subjective sense of presence, emotion recognition, and experienced emotions in auditory virtual environments. *CyberPsychol. Behav.* 6 181–188. 10.1089/109493103321640374 12804030

[B47] Willans T. Rivers S. Prasolova-Forland E. (2016). “Enactive emotion and presence in virtual environments,” in *Emotions, Technology, and Behaviors. A volume in Emotions and Technology*, eds Tettegah S. Esplege D. (Amsterdam: Elsevier), 181–210.

[B48] Wilson C. (2018). Is it love or loneliness? Exploring the impact of everyday digital technology use on the wellbeing of older adults. *Ageing Soc.* 38 1307–1331. 10.1017/S0144686X16001537

[B49] Winsler A. Ducenne L. Koury A. (2011). Singing one’s way to self-regulation: the role of early music and movement curricula and private speech. *Early Educ. Dev.* 22 274–304. 10.1080/10409280903585739

[B50] Wolf K. Reinhardt J. Funk M. (2018). “Virtual exhibitions: what do we win and what do we lose?,” in *Proceedings of the Conference on Electronic Visualisation and the Arts* (Slough: BCS Learning & Development Ltd), 79–86. 10.14236/ewic/EVA2018.15

